# High Resolution Size Analysis of Fetal DNA in the Urine of Pregnant Women by Paired-End Massively Parallel Sequencing

**DOI:** 10.1371/journal.pone.0048319

**Published:** 2012-10-31

**Authors:** Nancy B. Y. Tsui, Peiyong Jiang, Katherine C. K. Chow, Xiaoxi Su, Tak Y. Leung, Hao Sun, K. C. Allen Chan, Rossa W. K. Chiu, Y. M. Dennis Lo

**Affiliations:** 1 Centre for Research into Circulating Fetal Nucleic Acids, Department of Chemical Pathology, Li Ka Shing Institute of Health Sciences, The Chinese University of Hong Kong, Shatin, New Territories, Hong Kong SAR, China; 2 Centre for Research into Circulating Fetal Nucleic Acids, Department of Obstetrics and Gynaecology, Li Ka Shing Institute of Health Sciences, The Chinese University of Hong Kong, Shatin, New Territories, Hong Kong SAR, China; VU University Medical Center, The Netherlands

## Abstract

**Background:**

Fetal DNA in maternal urine, if present, would be a valuable source of fetal genetic material for noninvasive prenatal diagnosis. However, the existence of fetal DNA in maternal urine has remained controversial. The issue is due to the lack of appropriate technology to robustly detect the potentially highly degraded fetal DNA in maternal urine.

**Methodology:**

We have used massively parallel paired-end sequencing to investigate cell-free DNA molecules in maternal urine. Catheterized urine samples were collected from seven pregnant women during the third trimester of pregnancies. We detected fetal DNA by identifying sequenced reads that contained fetal-specific alleles of the single nucleotide polymorphisms. The sizes of individual urinary DNA fragments were deduced from the alignment positions of the paired reads. We measured the fractional fetal DNA concentration as well as the size distributions of fetal and maternal DNA in maternal urine.

**Principal Findings:**

Cell-free fetal DNA was detected in five of the seven maternal urine samples, with the fractional fetal DNA concentrations ranged from 1.92% to 4.73%. Fetal DNA became undetectable in maternal urine after delivery. The total urinary cell-free DNA molecules were less intact when compared with plasma DNA. Urinary fetal DNA fragments were very short, and the most dominant fetal sequences were between 29 bp and 45 bp in length.

**Conclusions:**

With the use of massively parallel sequencing, we have confirmed the existence of transrenal fetal DNA in maternal urine, and have shown that urinary fetal DNA was heavily degraded.

## Introduction

Genome-wide analysis of cell-free fetal DNA in maternal plasma has been achieved with the use of massively parallel sequencing (MPS) [1]. This development has allowed an accurate noninvasive diagnosis of fetal chromosomal abnormalities [2]–[5]. In addition to genetic analysis, the physical property of plasma DNA has been revealed by MPS. Cell-free DNA molecules in plasma are mostly associated with the nucleosomes, with the fetal-derived DNA molecules generally shorter than those derived from the mother [6]. In this study, we have applied MPS for the investigation of cell-free fetal DNA molecules in another type of clinically important body fluid, i.e., maternal urine.

Cell-free DNA in urine is mainly derived from two sources, i.e., the locally degraded DNA from the urinary tract, and the transrenal DNA excreted from the plasma [7]. The phenomenon of transrenal DNA passage has been demonstrated in various clinical scenarios. In the urine of female patients receiving blood transfusion, the presence of donor-derived male DNA has been reported [8]. Similarly, with the use of a sex-mismatched hematopoietic stem cell transplantation model in which most of the plasma DNA of the recipients was found to possess a donor-derived genotype, donor-derived DNA was also detectable in the recipients’ urine [9]. In nasopharyngeal carcinoma patients, the transrenal excretion of Epstein-Barr virus DNA from the plasma into the urine has been demonstrated [10].

With regard to pregnancy, fetal DNA is cleared rapidly from maternal plasma following delivery, with an apparent half-life of 16 min [11]. One possible clearance mechanism is the transrenal excretion of fetal DNA into maternal urine. However, inconsistent findings concerning the existence of fetal DNA in maternal urine have been reported. Botezatu *et al* have detected male fetal DNA in eight of ten first-trimester maternal urine samples [8]. However, Al-Yatama *et al* and Majer *et al* have showed that the sensitivities of urinary fetal DNA detection were only 38% and 32%, respectively [12], [13]. Urinary fetal DNA was undetectable in three other reports [14]–[16]. Subsequently, researchers have showed that the detection rate of urinary fetal DNA was enhanced by shortening the amplicons of the PCR assays, suggesting that fetal DNA fragments are short in length [17], [18]. However, a systematic study of the high resolution size profile of fetal DNA in maternal urine has not been performed.

The lack of knowledge on the concentration and the integrity of fetal DNA in maternal urine has hampered the development of the field. In fact, the presumably low concentration of the heavily degraded transrenal fetal DNA would make it difficult to be detected by PCR. In this study, we have utilized the MPS approach to precisely measure the fractional concentration of fetal DNA molecules in maternal urine, as well as to determine their size distribution profiles at high resolution.

## Materials and Methods

### Ethical Statement

The study was approved by the Clinical Research Ethics Committee of The Chinese University of Hong Kong. All subjects were recruited with written informed consent.

### Study Participants and Sample Collection

We recruited pregnant women from the Department of Obstetrics and Gynaecology, Prince of Wales Hospital, Hong Kong. Only women with singleton pregnancies were recruited. For 14 pregnant women, we collected catheterized urine immediately before cesarean delivery and at 24 h after delivery. Spontaneously voided urine was additionally collected from one of the women at one month post-delivery. We also collected peripheral blood and delivered placental tissues from these women. For another nine pregnant women, we collected spontaneously voided urine before delivery. Spontaneously voided urine samples were also collected from one male and one non-pregnant female.

### Sample Processing

Urine was collected into sterile plain bottles, and was mixed with EDTA, pH 8.0 (Ambion), to a final concentration of 10 mmol/L in order to inhibit nuclease activities. We centrifuged the urine at 1600 *g* for 10 min at 4°C, and filtered the supernatant through a 5-µm filter (Millipore). Peripheral blood was collected into EDTA tubes, and was centrifuged at 1600 *g* for 10 min at 4°C. The plasma portion was recentrifuged at 16,000 *g* for 10 min at 4°C. The blood cell portion was recentrifuged at 2500 *g* for 5 min and any residual plasma was removed. All samples were stored at −80°C until DNA extraction.

### DNA Extraction

We extracted DNA from 10 to 40 mL of filtered urine. We mixed 15 mL of 6 M guanidine isothiocyanate (Sigma-Aldrich) and 1 mL of resin (Wizard *Plus* Minipreps DNA Purification System, Promega) for every 10 mL of urine. The mixture was incubated with gentle mixing for 2 h at room temperature. The DNA bound resin was then isolated and washed on minicolumns with the wash buffer provided in the Wizard *Plus* Minipreps DNA Purification System (Promega) according to the manufacturer’s instructions. DNA was eluted in 100 µL of water for every 10 mL of starting urine volume. We extracted DNA from one maternal plasma sample with the same protocol, with 20 mL of guanidine isothiocynate and 4 mL of resin added to 4 mL of plasma. DNA was isolated from the placental tissues and the blood cells with the QIAamp tissue kit (Qiagen) and the QIAamp blood kit (Qiagen) according to the manufacturer’s protocol.

### Real-time Quantitative PCR

We quantified urinary DNA with a real-time PCR assay directed toward the *leptin (LEP)* gene, with the primers 5′-CAGTCTCCTCCAAACAGAAAGTCA-3′ and 5′-CAGGATGGGGTGGAGCC-3′, and the fluorescent probe 5′-(VIC)CGGTTTGGACTTCA(MGBNFQ)-3′ (Applied Biosystems), where MGBNFQ is a minor groove-binding nonfluorescent quencher. The assay produced a 62-bp amplicon. The reaction was set up with TaqMan PCR Reagent Kit (Applied Biosystems) in a reaction volume of 50 µL using 600 nM of each primer and 200 nM of the probe. We used 5 µL of urinary DNA for quantification in an ABI 7300 real-time PCR system (Applied Biosystems). The reaction profile was 50°C for 2 min, 95°C for 10 min, and 45 cycles of 95°C for 15 s and 58°C for 1 min.

### SNP Genotyping

Fetal and maternal genomic DNA samples were extracted from the placental tissues and the maternal blood cells, respectively, and were genotyped for approximately 900,000 SNPs with the Genome-Wide Human SNP Array 6.0 (Affymetrix) according to the manufacturer’s instructions.

### DNA Sequencing

We constructed sequencing libraries by the Paired-End DNA Sample Prep Kit (Illumina) with the protocol previously developed for plasma DNA sequencing [19]. DNA libraries were sequenced with a Genome Analyzer IIx (Illumina) or a HiSeq 2000 (Illumina) using 36-bp×2 or 50-bp×2 format, respectively. For paired-end (PE) reads with inserted DNA fragments shorter than the sequenced length, adaptor-derived nucleotides were sequenced at the end of the reads. These “contaminated” adaptor sequences were trimmed from the reads before genomic alignment with the SOAP2 program (http://soap.genomics.org.cn).

For fetal DNA detection using the chromosome Y (chrY) approach, sequenced reads were aligned to the repeat-masked reference human genome (hg18) with no mismatch allowed. For all of the other analyses, sequenced reads were aligned to the non-repeat-masked reference human genome (hg18), allowing up to two nucleotide mismatches in either member of the PE reads. We included only PE reads that were uniquely aligned to a single location in the genome with a correct orientation. We removed all but one of the duplicated PE reads that showed identical start and end alignment locations in the genome. The length of the inserted DNA was inferred from the coordinates of the outermost nucleotides at the two ends of the sequenced fragments. We included DNA fragments with sizes from 20 bp to 600 bp.

### Statistical Analysis

Statistical analysis was performed with SigmaStat 3.5 (Systat Software). A difference with *P* values <0.05 was considered statistically significant.

## Results

### LEP DNA Concentrations in Maternal Urine

**Figure 1:**
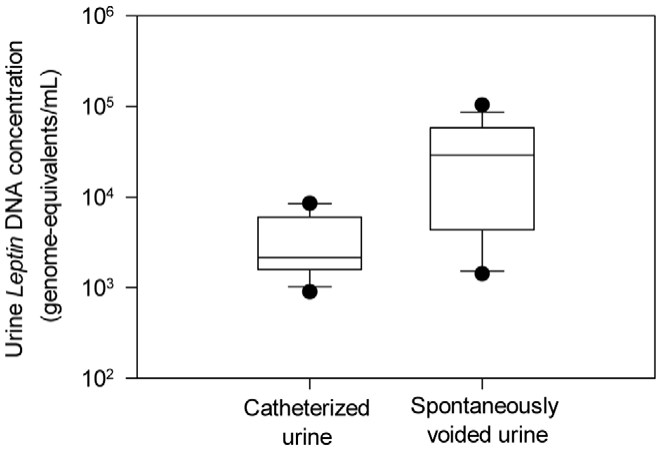
*LEP* DNA concentrations in the catheterized and spontaneously voided maternal urine samples. The lines inside the boxes denote the medians. The boxes mark the interval between the 25^th^ and 75^th^ percentiles. The whiskers denote the interval between the 10^th^ and 90^th^ percentiles. The circles mark the data points outside the 10^th^ and 90^th^ percentiles.

 We sought to determine if maternal urine collected through a catheter or by spontaneous voiding is more suitable for fetal DNA analysis. We measured total cell-free DNA concentrations in the catheterized and the spontaneously voided maternal urine by using a real-time quantitative PCR assay for the *LEP* gene. The *LEP* DNA concentrations in the spontaneously voided urine samples were significantly higher than those in the catheterized urine samples (Mann-Whitney Rank Sum test, *P*<0.05) (Figure 1). The relatively high *LEP* DNA level in the spontaneously voided urine, as compared with the catherized urine, was possibly due to the local release of maternal DNA from the renal epithelial cells during urine storage in the bladder. Since our purpose was to investigate transrenal fetal DNA in maternal urine, in order to reduce the interference from the background maternal DNA, we used catheterized maternal urine samples in the subsequent MPS study.

### PE Sequencing of Urinary Cell-free DNA

**Figure 2:**
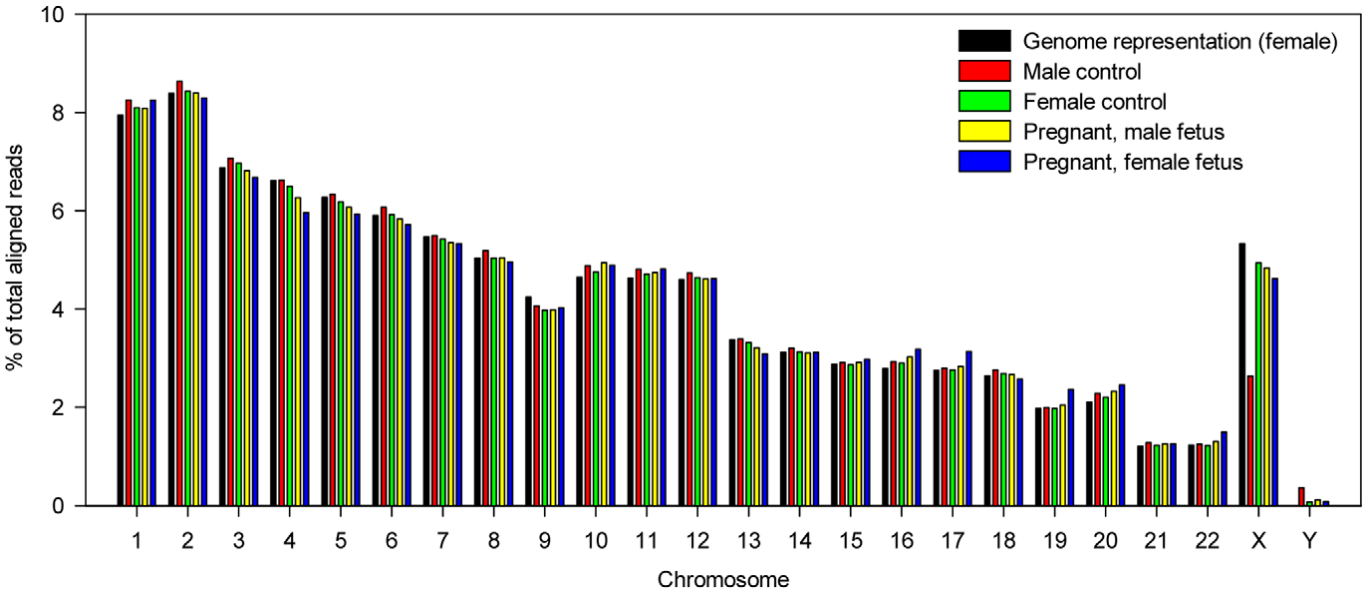
Genomic distribution urinary DNA fragments among the human chromosomes. The percentage of aligned PE reads per chromosome for the urine samples of a male control (red bars), a female control (green bars), a woman carrying male fetus (yellow bars) and a woman carrying female fetus (blue bars). The chromosomal representation as expected for the non-repeat-masked female diploid genome (black bars) is included for reference.

For MPS analysis, we studied urine samples obtained from one male, one non-pregnant female and seven pregnant women during the third trimester of pregnancy. On average, 58% of raw PE reads were mapped uniquely to the non-repeat-masked reference human genome with two or less nucleotide mismatches (Table S1). Figure 2 shows the distributions of aligned PE reads among the human chromosomes for the male and the female urine, as well as two of the seven maternal urine in which a male and a female fetus was involved. We performed linear regression analysis to compare the chromosomal distribution of the aligned reads against the expected genomic representation of each chromosome in the human genome. The R^2^ were over 0.95 for all of the controls and the seven maternal urine samples. The result hence demonstrated an even distribution of total urinary DNA molecules across the human genome.

### Fractional Fetal DNA Concentration in Maternal Urine: the chrY Approach

We investigated the use of chrY-derived DNA sequences as fetal-specific markers to estimate the fractional fetal DNA concentrations (fetal%) in the urine of women carrying male fetuses. We counted the number of PE reads that aligned perfectly to chrY and non-chrY of the repeat-masked reference human genome. In our previous studies using plasma DNA, we observed a small percentage of wrongly aligned chrY reads for female samples [2], [19]. Hence, we first estimated the false chrY-alignment rate in the case of urinary DNA. We measured the chromosomal percentages of chrY reads (Y%) in the urine of one male and one non-pregnant female, which were 0.216% and 0.035%, respectively (Table 1 and Table S2). We then calculated fetal% of the maternal urine samples with the correction of chrY false alignment using the equation as previously described [19]:
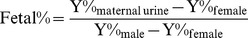
where Y% _maternal urine_ is the Y% of the maternal urine samples, Y% _female_ and Y% _male_ are the Y% of the female and male urine samples, which are 0.035% and 0.216%, respectively.

**Table 1:** Fractional fetal DNA concentrations in maternal urine as calculated by the chromosome Y approach.

	Case	Fetal sex	Y%	Fetal%^a^
Control	Male	−	0.216%	−
	Female	−	0.035%	−
Pregnant women	6849	M	0.045%	0.057%
	6918	M	0.070%	0.198%
	7401	M	0.041%	0.037%
	8542	M	0.037%	0.015%
	7413	F	0.042%	0.042%
	7418	F	0.050%	0.088%
	7482	F	0.041%	0.036%

a

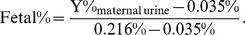

We measured fetal% in the urine of four women carrying male fetuses and three women carrying female fetuses. If fetal DNA was present in maternal urine, we expected a higher fetal% in the male-fetus group when compared to the female-fetus group. However, as shown in Table 1, the fetal% was completely overlapping between the two groups (range of male-fetus group: 0.015%–0.198%; range of female-fetus group: 0.036%–0.088%). Hence, the presence of urinary fetal DNA could not be ascertained by these data.

We reasoned that fetal% measurement based on the counting of chrY DNA fragments might be imprecise for urinary DNA study. Since urinary DNA fragments are presumably ultra short in length, the sequenced contents of the two members of the PE reads would be mostly overlapped and resembled single-reads (SR) when performing genome alignment. To estimate if the chance of false chrY-alignment would be increased for SR alignment, we retrieved the PE sequencing data of four maternal plasma samples involving female fetuses from our previous studies [2], [3]. SR alignment was simulated by re-aligning the data with only one member of the PE reads. The median Y% due to misalignment for the SR result was 0.0083%, which was 84% higher than that of the PE alignment (0.0045%). Hence, while Y% measurement was robust for plasma DNA molecules [19], in which a majority of them were longer than 100 bp [6], [19], it might not be reliable for urine study in terms of alignment accuracy.

### Fractional Fetal DNA Concentration in Maternal Urine: the SNP Approach

We next used a SNP-based approach to measure fetal% in maternal urine. To identify fetal DNA fragments, we utilized informative SNPs in which the mother was homozygous (AA) and the fetus was heterozygous (AB) for the genotype. The numbers of PE reads that contained the fetal-specific allele (the B allele) as well as the allele shared by the mother and the fetus (the A allele) were counted. The proportion of DNA sequences containing the fetal-specific allele was calculated by the equation:
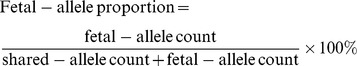



Fetal DNA was considered to be present if the fetal-allele proportion was above the limit of detection (LOD) (Methods S1). For the maternal urine samples with fetal-allele proportion >LOD, fetal% was further calculated by the equation:
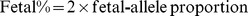



Fetal DNA was detected in five of the seven maternal urine samples, with the fetal% ranging from 1.92% to 4.73% (Table 2 and Table S3). We also analyzed urine samples collected from these pregnant women at 24 h after delivery. Fetal DNA became undetected in all except one post-delivery urine sample (Table 2). For case 8542, the fetal% decreased from 3.85% to 1.38% after delivery. To investigate if urinary fetal DNA might persist in this case, an additional urine sample was collected from the woman one month after delivery. Fetal DNA was no longer detected in this sample (Table 2). Hence, by using the SNP approach, we have confirmed that cell-free fetal DNA was present in maternal urine, and disappeared after delivery.

**Table 2:** Fractional fetal DNA concentrations in maternal urine as calculated by the SNP approach.

Case	Fetal%^a^		
	Before delivery	24 hours after delivery	1 month after delivery
6849	1.92%	<LOD^b^	−
6918	4.70%	<LOD	−
7401	<LOD	<LOD	−
7413	4.39%	<LOD	−
7418	<LOD	<LOD	−
7482	4.73%	<LOD	−
8542	3.85%	1.38%	<LOD

a

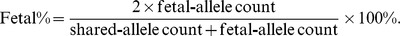

b LOD, limit of detection. Samples with proportion of fetal-allele fragments lower than the LOD were marked as <LOD (Table S3 and Methods S1).

### Size Distribution of Cell-free DNA in Maternal Urine

We first compared the size distributions of cell-free DNA molecules in the plasma and urine samples collected from case 6918. The sizes of fetal DNA were deduced from the PE reads with the fetal-specific alleles. The maternal DNA sizes were deduced from the PE reads of the shared-allele because over 95% of these reads were maternally derived (Table 2).

**Figure 3:**
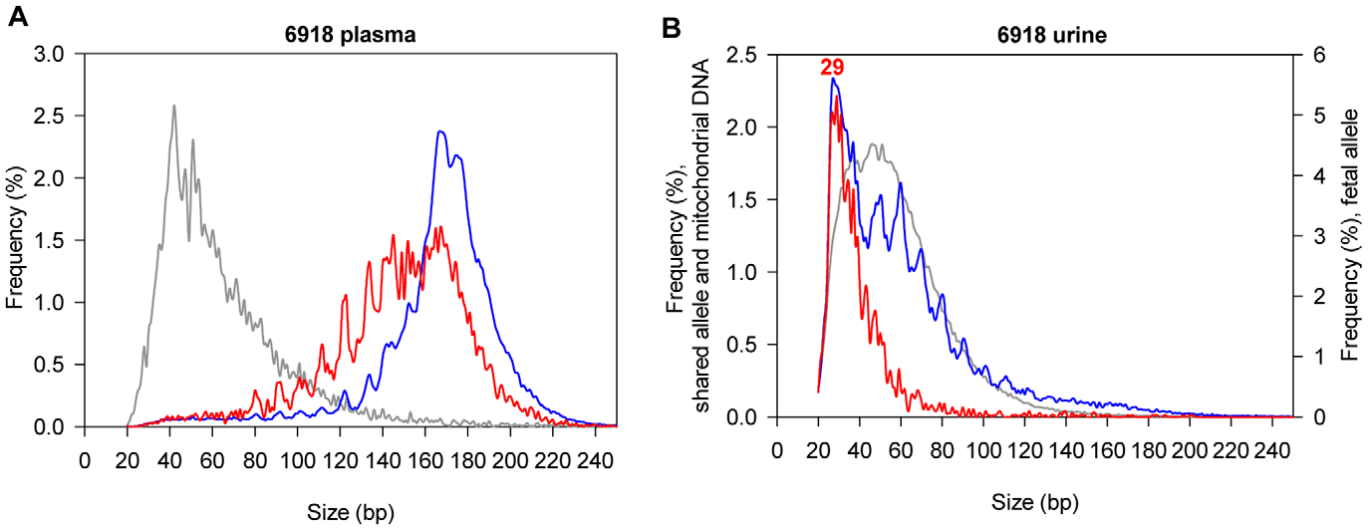
Comparison of size distributions between plasma and urinary DNA fragments. Size distributions of fetal DNA (red curves), maternal DNA (blue curves) and mitochondrial DNA (grey curves) in (A) maternal plasma and (B) maternal urine of case 6918. The number denotes the DNA size of the predominant urinary fetal DNA peak.

**Figure 4:**
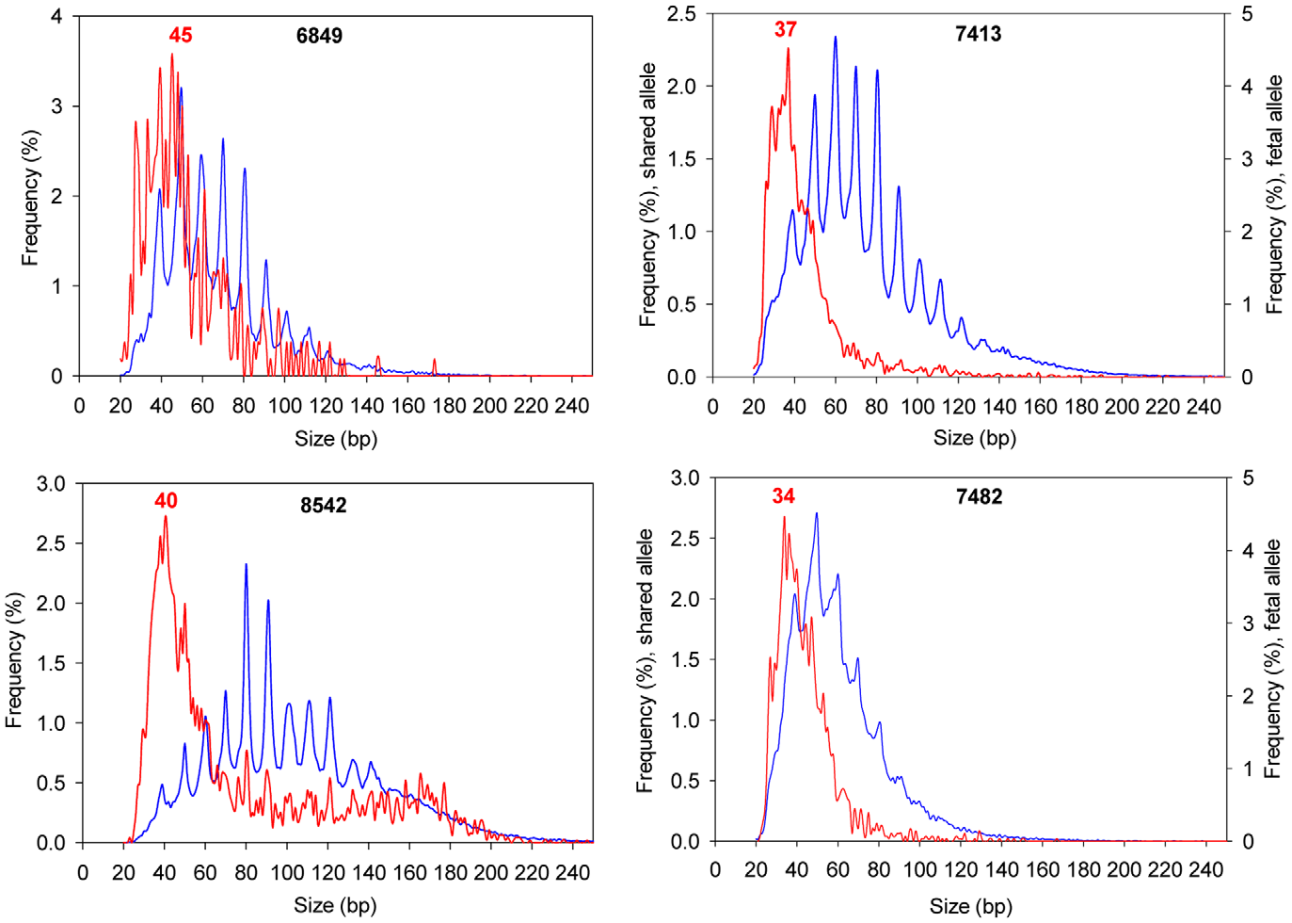
Size distribution of DNA fragments in maternal urine. Size distributions of fetal DNA (red curves) and maternal DNA (blue curves) in the maternal urine in cases 6849, 7413, 7482 and 8542. The numbers denote the DNA sizes of the predominant urinary fetal DNA peak in each sample.

As shown in Figure 3A, the size distribution of plasma DNA fragments was consistent with the previous studies [6]. The maternal DNA fragments had a most frequent fragment size of 166 bp, and fetal DNA was generally shorter than maternal DNA [20]. On the other hand, in the maternal urine, the maternal DNA fragments were much shorter than those found in the plasma (Figure 3B). A majority of maternal DNA fragments in the urine were less than 100 bp, with a 10-bp periodicity of peaks observed. More importantly, we found that fetal DNA fragments were very short in maternal urine, and exhibited a predominant peak at 29 bp (Figure 3B). Figure 4 shows the size distributions of the other four maternal urine samples in which fetal DNA was detected (Table 2). In general, the size distributions of urinary fetal DNA were similar among all of the five maternal urine samples (Figures 3B and 4), with predominant peaks observed at the DNA sizes ranging from 29 bp to 45 bp.

In contrast to nuclear DNA, the size distributions of mitochondrial DNA were similar between the plasma and the urine samples (Figure 3).

Based on the result of size distribution, we concluded that cell-free DNA molecules in urine were more degraded than those in plasma. In addition, fetal DNA fragments were very short in maternal urine.

## Discussion

We have confirmed in this study that fetal DNA is indeed present in maternal urine. By using MPS, we were able to detect cell-free fetal DNA in five of the seven (71%) maternal urine samples. Fetal DNA constituted a median of 3.85% of total DNA in maternal urine, which was very low when compared to the fetal% of maternal plasma with matched gestations, i.e., 20.4% [21]. The observed urinary fetal DNA molecules were most probably derived from the transrenal excretion of fetal DNA in maternal plasma. This is based on the observation that fetal DNA was absent in most of the maternal urine samples 24 h after delivery, and was consistent with a previous report of the rapid clearance of fetal DNA from maternal plasma post-delivery [11]. In this study, we have only studied one post-delivery time point, i.e., at 24 h after delivery. To further investigate the kinetics of the transrenal clearance of plasma fetal DNA, it would be interesting to compare fetal% in plasma and urine collected serially after delivery.

In this study, we have also determined the integrity of cell-free DNA in maternal urine. Fetal DNA fragments were very short, with predominant peaks at the sizes of 29 bp to 45 bp (Figures 3 and 4). The low integrity of urinary fetal DNA may be caused by several factors. In maternal plasma, only a subpopulation of fetal DNA fragments with sizes smaller than the kidney filter [22] could escape into the urine. After reaching the urine, fetal DNA may be subjected to heavy degradation because nuclease activity in urine was reported to be 4-fold higher than that in plasma [23]. In addition, while fetal DNA is coiled around nucleosomes in maternal plasma [6], it remains to be explored if urinary fetal DNA is being protected by associating with any biomolecules.

Maternal-derived urinary DNA molecules may be released both transrenally and locally. In our previous study using a hematopoietic stem cell transplantation model, while 76% of the recipient’s plasma DNA molecules were found to be donor-derived, such donor DNA contributed 38% of the cell-free DNA in the recipient’s urine [9]. This finding suggests that a proportion of the cell-free DNA molecules in urine are derived transrenally from plasma. Renal epithelial cells may represent another source of cell-free DNA in urine. The size distribution of urinary maternal DNA showed a 10-bp periodicity (Figures 3 and 4), which resembled the pattern of nuclease cleavage of nucleosome-bound plasma DNA fragments [6], [19]. Hence, such urinary maternal DNA molecules may be released locally from epithelial cells through apoptosis [24]. The relative contribution of cell-free urinary DNA by different biological sources may be further investigated, possibly by using a renal transplantation model [25], [26]. In general, the total cell-free DNA molecules in urine were less intact than those in plasma (Figure 3). This is possibly due to the potent nuclease activity in urine [23]. In contrast to nuclear DNA, we found that the size distributions of mitochondrial DNA were largely similar between the plasma and the urine (Figure 3). One of the reasons may be due to the circular structure of mitochondrial DNA molecules, which may make them less susceptible to degradation by exonucleases [27].

Our finding of size distribution of fetal DNA in maternal urine might explain the controversial results concerning transrenal fetal DNA in the previous reports. To detect fetal DNA in maternal urine, most of these reports used PCRs with amplicon sizes of 63 bp to 393 bp [8], [12]–[17], which might not be efficient to pick up the extremely short fetal DNA templates. By testing PCRs with amplicons ranging from 25 bp to 88 bp, Shekhtman *et al* have shown that the detection rate of fetal DNA in maternal urine was the highest for a PCR amplicon size of 25 bp [18]. In the current study, we have used the PE format of MPS to study cell-free DNA in urinary supernatants. The system is able to detect extremely short DNA fragments (≥20 bp) and measure the length of individual fragments at single-base resolution. Hence, we have for the first time revealed the high resolution size distributions of fetal- and maternal-derived DNA in maternal urine.

In conclusion, this study has provided solid evidence for the existence of cell-free fetal DNA in maternal urine. Biologically, the result suggests that renal clearance would be one of the mechanisms to eliminate fetal DNA from maternal plasma. Clinically, the findings may open up new possibilities for non-invasive diagnostic applications. Since the transrenal passage of fetal DNA depends on the kidney filter, one of the immediate applications would to study the kidney-associated pathologies, such as preeclampsia [28]. In addition to pregnancy, the result of this study may have implication for other clinical situations such as cancer diagnosis [29]–[31] and renal transplantation monitoring [25], [32].

## Supporting Information

Table S1 **Sequence alignment result of urinary DNA PE sequencing.**
(DOCX)

Table S2 **Calculation of Y% in the urine of controls and pregnant women.**
(DOCX)

Table S3 **Calculation of fractional fetal DNA concentration in maternal urine by the SNP approach.**
(DOCX)

Methods S1 **Determination of the limit of detection (LOD) of MPS for measuring fractional fetal DNA concentration in maternal urine.**
(DOCX)
