# Supplementary material for: High Resolution Size Analysis of Fetal DNA in the Urine of Pregnant Women by Paired-End Massively Parallel Sequencing
Source: PLoS One. 2012 Oct 31;7(10):e48319. doi: 10.1371/journal.pone.0048319 (PMC3485143; doi:10.1371/journal.pone.0048319)
Supplement: Table S1 — Sequence alignment result of urinary DNA PE sequencing. (DOCX) [file pone.0048319.s001.docx]

**Table S1.** Sequence alignment result of urinary DNA PE sequencing.

|  | **Case** | **Raw PE reads** | **Aligned PE reads^a^** | **Aligned %** |
| --- | --- | --- | --- | --- |
| Control | Male | 35,314,267 | 23,972,018 | 67.88% |
|  | Female | 38,531,170 | 23,253,008 | 60.35% |
| Pregnant women | 6849 | 41,335,115 | 22,225,812 | 53.77% |
|  | 6918 | 150,634,946 | 71,276,970 | 47.32% |
|  | 7401 | 37,331,678 | 28,276,999 | 75.75% |
|  | 7413 | 168,529,646 | 114,972,336 | 68.22% |
|  | 7418 | 33,709,891 | 24,275,331 | 72.01% |
|  | 7482 | 70,051,046 | 18,123,832 | 25.87% |
|  | 8542 | 159,649,827 | 74,322,117 | 46.55% |
| ^a^PE reads that uniquely aligned to the non-repeat-masked reference human genome (hg18) allowing up to 2 nucleotide mismatches. | | | | |
