# Supplementary material for: High Resolution Size Analysis of Fetal DNA in the Urine of Pregnant Women by Paired-End Massively Parallel Sequencing
Source: PLoS One. 2012 Oct 31;7(10):e48319. doi: 10.1371/journal.pone.0048319 (PMC3485143; doi:10.1371/journal.pone.0048319)
Supplement: Table S2 — Calculation of Y% in the urine of controls and pregnant women. (DOCX) [file pone.0048319.s002.docx]

**Table S2.** Calculation of Y% in the urine of controls and pregnant women.

|  | **Case** | **Fetal sex** | **Non-Y count** | **Y count** | **Y%^a^** |
| --- | --- | --- | --- | --- | --- |
| Control | Male | - | 10,490,647 | 22,715 | 0.216% |
|  | Female | - | 9,856,295 | 3,402 | 0.035% |
| Pregnant women | 6849 | M | 10,016,180 | 4,501 | 0.045% |
|  | 6918 | M | 35,387,986 | 24,927 | 0.070% |
|  | 7401 | M | 12,477,617 | 5,146 | 0.041% |
|  | 8542 | M | 31,905,193 | 11,908 | 0.037% |
|  | 7413 | F | 52,561,170 | 22,194 | 0.042% |
|  | 7418 | F | 11,119,870 | 5,611 | 0.050% |
|  | 7482 | F | 22,644,919 | 9,294 | 0.041% |
| ^a^ Y% = (Y count) / (non-Y count + Y count) x 100% | | | | | |
