# Supplementary material for: High Resolution Size Analysis of Fetal DNA in the Urine of Pregnant Women by Paired-End Massively Parallel Sequencing
Source: PLoS One. 2012 Oct 31;7(10):e48319. doi: 10.1371/journal.pone.0048319 (PMC3485143; doi:10.1371/journal.pone.0048319)
Supplement: Table S3 — Calculation of fractional fetal DNA concentration in maternal urine by the SNP approach. (DOCX) [file pone.0048319.s003.docx]

**Table S3**. Calculation of fractional fetal DNA concentration in maternal urine by the SNP approach.

| **Case** | **Urine collection time** | **Shared allele count^a^** | **Fetal allele count^a^** | **Fetal-allele proportion^b^** | **Fetal%^d^** |
| --- | --- | --- | --- | --- | --- |
| 6849 | Pre-delivery | 55,005 | 533 | 0.96% | 1.92% |
|  | 24h post-delivery | 82,163 | 380 | 0.46% (<LOD^c^) |  |
| 6918 | Pre-delivery | 132,183 | 3,181 | 2.35% | 4.70% |
|  | 24h post-delivery | 38,059 | 187 | 0.49% (<LOD) |  |
| 7401 | Pre-delivery | 75,665 | 308 | 0.41% (<LOD) |  |
|  | 24h post-delivery | 91,288 | 363 | 0.40% (<LOD) |  |
| 7413 | Pre-delivery | 302,283 | 6,788 | 2.20% | 4.39% |
|  | 24h post-delivery | 90,283 | 340 | 0.38% (<LOD) |  |
| 7418 | Pre-delivery | 59,067 | 268 | 0.45% (<LOD) |  |
|  | 24h post-delivery | 105,113 | 450 | 0.43% (<LOD) |  |
| 7482 | Pre-delivery | 115,883 | 2,805 | 2.36% | 4.73% |
|  | 24h post-delivery | 69,321 | 315 | 0.45% (<LOD) |  |
| 8542 | Pre-delivery | 244,761 | 4,807 | 1.93% | 3.85% |
|  | 24h post-delivery | 337,461 | 2,343 | 0.69% | 1.38% |
|  | 1 month post-delivery | 156,811 | 905 | 0.57% (<LOD) |  |
| ^a^ Shared allele, allele shared by the mother and the fetus; fetal allele, allele specific to the fetus.  ^b^Fetal-allele proportion = fetal-allele count / (shared-allele count + fetal-allele count) x 100%.  ^c^LOD, limit of detection, which was equal to 0.59% (Methods S1).  ^d^Fetal% = 2 x fetal-allele proportion (%). | | | | | |
