# Supplementary material for: High Resolution Size Analysis of Fetal DNA in the Urine of Pregnant Women by Paired-End Massively Parallel Sequencing
Source: PLoS One. 2012 Oct 31;7(10):e48319. doi: 10.1371/journal.pone.0048319 (PMC3485143; doi:10.1371/journal.pone.0048319)
Supplement: Methods S1 — Determination of the limit of detection (LOD) of MPS for measuring fractional fetal DNA concentration in maternal urine. (DOCX) [file pone.0048319.s004.docx]

**Methods S1**

**To determine the limit of detection (LOD) of MPS for measuring fractional fetal DNA concentration in maternal urine**

In the current study, we identified fetal-derived DNA fragments by using the SNP alleles that were specific to the fetus. We reasoned that the errors due to (i) microarray SNP genotyping, and (ii) massively parallel sequencing (MPS) and sequence alignment, would lead to the false identification of DNA fragments as fetal-derived, which in turn would affect the accuracy of fetal DNA detection. We therefore determined the proportion of fetal-allele sequences that were misidentified due to these two errors.

1. *To determine the proportion of fetal-allele sequences misidentified due to microarray SNP genotyping errors*

We used the MPS method to validate the fetal and maternal SNP genotypes as determined by the microarray for case 8542.

Sequencing libraries were prepared from the fetal and maternal genomic DNA by a TruSeq DNA Sample Preparation Kit (Illumina) according to the manufacturer’s instructions, and sequenced with a HiSeq 2000 (Illumina) using 100bp x 2 Paired-End (PE) format. Three sequencing lanes were used for each genomic sample in order to achieve an average sequencing depth of over 10-fold. Sequenced reads were aligned to the non-repeat-masked reference human genome (hg18) allowing up to 2 nucleotide mismatches in either member of the PE reads. Genomic positions of the SNPs that were represented in the Genome-Wide Human SNP Array 6.0 (Affymetrix) were retrieved, and genotyping was performed when these SNP positions were covering by ≥8 PE reads. The SNVMix model [1] was used to determine the homozygous and heterozygous SNP genotypes by using the aligned reads with a confidence of >99.9%.

As shown in Table I, around 1.4% of the SNPs that were covered by both MPS and the microarray showed inconsistent genotypes. We speculated that the discordance was mostly contributed by the genotyping error of the microarray.

Table I. Comparison of SNP genotyping result by MPS and microarray.

| **Case 8542** | **Genotyping by MPS** | |  | **MPS vs microarray genotyping result** | | |
| --- | --- | --- | --- | --- | --- | --- |
|  | Average sequencing depth | No. of SNPs overlapped  with microarray |  | Concordant  SNPs | Discordant  SNPs | Discordance  % |
| Fetal genomic DNA | 12x | 652,312 |  | 642,679 | 9,633 | 1.48% |
| Maternal genomic DNA | 16x | 776,999 |  | 766,682 | 10,317 | 1.33% |

We selected the SNPs that showed consistent genotyping result between MPS and the microarray, i.e., the “concordant” SNPs, for the calculation of fetal-allele proportion. Three maternal urine samples collected at different time points for the case 8542 were analyzed. Informative SNPs in which the mother was homozygous (AA) and the fetus was heterozygous (AB) for the genotypes were used. The numbers of reads containing the shared allele (the A allele) and the fetal alleles (the B allele) were counted. The fetal-allele proportions were further calculated as described in Table II. As shown in Table II, by comparing the fetal-allele proportions calculated using the “concordant” and the “microarray” SNP groups, we found that the microarray genotyping error has led to an overestimation of fetal-allele sequences. An average of 0.42% of fetal-allele proportion was introduced due to the error.

Table II. Estimation of fetal allele proportion introduced by microarray genotyping error.

| **Case 8542**  **(Urine collection time)** | **SNP group^a^** | **Shared-allele count** | **Fetal-allele count** | **Fetal-allele proportion^b^** | **Overestimated fetal-allele proportion due to microarray error^c^** |
| --- | --- | --- | --- | --- | --- |
| Before delivery | Microarray | 244,761 | 4,807 | 1.93% | 0.43% |
|  | Concordant | 145,314 | 2,200 | 1.49% |  |
| 24 hours  after delivery | Microarray | 337,461 | 2,343 | 0.69% | 0.42% |
|  | Concordant | 207,764 | 567 | 0.27% |  |
| 1 month  after delivery | Microarray | 156,811 | 905 | 0.57% | 0.42% |
|  | Concordant | 98,604 | 156 | 0.16% |  |
| ^a^ Microarray, group of informative SNPs in which the genotypes were solely detected by the microarray. Concordant, group of informative SNPs in which the genotypes were consistent between MPS and the microarray. | | | | | |
| ^b^Fetal allele proportion = fetal allele count / (shared allele count + fetal allele count) x 100% | | | | | |
| ^c^ The overestimated percentage = feta-allele proportion_Microarray SNPs_ – fetal-allele proportion_Concordant SNPs_ | | | | | |

1. *To determine the proportion of fetal-allele sequences misidentified due to MPS and alignment errors*

We included only the “concordant” SNPs in this part of the study because their genotypes were largely free of microarray errors. To estimate the MPS and sequence alignment errors, we selected the SNPs in which the mother and the fetus were homozygous for the same allele (AA). We then counted the number of reads of the true allele (the A allele) and the unexpected allele (all non-A alleles). As shown in Table III, the average proportion of the unexpected-allele sequences was 0.17% among the three maternal urine samples. Based on this result, we estimated that during fetal DNA identification, 0.17% of DNA sequences were falsely classified as fetal-derived due to the MPS and alignment errors.

Table III. Calculation of unexpected-allele sequence proportion.

| **Case 8542**  **(Urine collection time)** | **True-allele count** | **Unexpected-allele count** | **Proportion of unexpected-allele DNA^a^** |
| --- | --- | --- | --- |
| Before delivery | 837,545 | 1,640 | 0.20% |
| 24 hours after delivery | 1,189,421 | 1,997 | 0.17% |
| 1 month after delivery | 569,468 | 910 | 0.16% |
| ^a^Proportion of unexpected-allele DNA =  unexpected-allele count / (true-allele count + unexpected allele count) x 100% | | | |

1. *Limit of detection of fetal DNA in maternal urine*

The sum of misidentified fetal-allele DNA proportion due to the microarray genotyping error (i.e., 0.42%) and MPS and alignment error (i.e., 0.17%) was 0.59%. Hence, we defined this percentage as the LOD of fetal DNA detection. Fetal DNA was considered to be present in the maternal urine sample when the fetal-allele DNA proportion was above 0.59%.

Reference:

1. Goya R, Sun MG, Morin RD, Leung G, Ha G, et al. SNVMix: predicting single nucleotide variants from next-generation sequencing of tumors. Bioinformatics 26: 730-736.
